# Preparation, Optimization and Activity Evaluation of PLGA/Streptokinase Nanoparticles Using Electrospray

**DOI:** 10.15171/apb.2017.017

**Published:** 2017-04-13

**Authors:** Nasrin Yaghoobi, Reza Faridi Majidi, Mohammad ali Faramarzi, Hadi Baharifar, Amir Amani

**Affiliations:** ^1^Department of Medical Nanotechnology, School of Advanced Technologies in Medicine, Tehran University of Medical Sciences, Tehran, Iran.; ^2^Department of Pharmaceutical Biotechnology, Faculty of Pharmacy, Tehran University of Medical Sciences, Tehran, Iran.; ^3^Medical Biomaterials Research Center (MBRC), Tehran University of Medical Sciences, Tehran, Iran.

**Keywords:** PLGA nanoparticles, SK, Size distribution, ANNs, Electrospray

## Abstract

***Purpose:*** PLGA nanoparticles (NPs) have been extensively investigated as carriers of different drug molecules to enhance their therapeutic effects or preserve them from the aqueous environment. Streptokinase (SK) is an important medicine for thrombotic diseases.

***Methods:*** In this study, we used electrospray to encapsulate SK in PLGA NPs and evaluate its activity. This is the first paper which investigates activity of an electrosprayed enzyme. Effect of three input parameters, namely, voltage, internal diameter of needle (nozzle) and concentration ratio of polymer to protein on size and size distribution (SD) of NPs was evaluated using artificial neural networks (ANNs). Optimizing the SD has been rarely reported so far in electrospray.

***Results:*** From the results, to obtain lowest size of nanoparticles, ratio of polymer/enzyme and needle internal diameter (ID) should be low. Also, minimum SD was obtainable at high values of voltage. The optimum preparation had mean (SD) size, encapsulation efficiency and loading capacity of 37 (12) nm, 90% and 8.2%, respectively. Nearly, 20% of SK was released in the first 30 minutes, followed by cumulative release of 41% during 72 h. Activity of the enzyme was also checked 30 min after preparation and 19.2% activity was shown.

***Conclusion:*** Our study showed that electrospraying could be an interesting approach to encapsulate proteins/enzymes in polymeric nanoparticles. However, further works are required to assure maintaining the activity of the enzyme/protein after electrospray.

## Introduction

The body's natural homeostasis systems control development of blood clots in the bloodstream. Disruption of homeostasis results in stroke, pulmonary embolism, deep vein thrombosis and acute myocardial infarction.^1^ The disease may be treated by administration of intravenous thrombolytic agents such as streptokinase (SK). SK is an extracellular enzyme produced by different strains of β-hemolytic streptococci.^2^ Its molar mass is 47 kDa and contains 414 amino acid residues.^3^

In drug delivery applications, polymeric nano‏-carriers are often considered as interesting delivery systems due to their biodegradability, biocompatibility, minimum antigenic properties, and ease of preparation.^4^ Poly (lactic-co-glycolic acid) (PLGA) is a polyester with fascinating characteristics such as solubility in various solvents and being approved by food and drug administration (FDA).^5-7^ Various methods including emulsion polymerization and solvent evaporation have been used to encapsulate protein based drugs in polymers such as PLGA.^8-10^ The main problems with many of these methods are production of broad distribution of particle size, drug exposure to organic solvents and low encapsulation efficiency.^11,12^

Electrospray or electro hydrodynamic atomization (EHDA) is a method in which liquid solution is atomized by an applied electric field. Solution is pumped with a constant flow rate by using a syringe pump. A positive voltage is applied to needle so that electrical field overcomes surface tension of droplets that are made at the tip of the needle. Consequently, nano- or micro-particles are generated on the collector which connected to negative electrode or earth.^13^ This fairly new method produces polymeric nano- or micro particles^12^ from polymers such as PLGA^14-16^ and chitosan.^17,18^ Advantages of this approach include capability of controlling morphology and size of the particles, producing nearly mono dispersed particles and obtaining high encapsulation efficiency (EE), with minimum unfavorable effects on the active ingredients (e.g. denaturation) during the process.^11^

In this work, the effect of three factors, namely, needle ID, applied voltage and ratio of polymer to enzyme was evaluated on the average size and size distribution of particles, using artificial neural networks (ANNs). Previous studies have shown that size of nanoparticles can be controlled in electrospray by changing parameters like voltage, rate of injection, and collecting distance.^11^ However, works on size distribution are very rare. Our previous work showed that PLGA nanoparticles with minimum polymer concentration and collecting distance have minimum size distribution regardless of value of flow rate.^19^ The only other paper on size distribution of prepared particles using electrospray is about microparticle of polylactic acid (PLA). The report compares the effect of concentration, voltage, nozzle to collector distance, flow rate and nozzle distance on size and size distribution of generated micro particles using limited number of samples in a one-factor-at-a-time approach. Limited samples were prepared and used for data generation in the work.^12^ Nevertheless, electrospray, similar to electrospinning, is a complex and multi-variant process.^11^ Thus, predicting the physicochemical properties of prepared nanoparticles, using one-factor-at-a-time is not feasible. Therefore, applying a modeling method to find the parameters affecting size and size distribution in this method appears inevitable. Artificial neural networks (ANNs) which mimic neural brain could be a proper tool to analyze the data here.

Furthermore, a limited number of works have so far tried to encapsulate a protein in an electrospray procedure. Of which, the only one we could find reporting bioactivity of the protein, was encapsulation of bovine serum albumin (BSA) in PLGA microparticles. The antigenic properties of BSA remained fairly intact after electrospray.^20^ This work is evaluating the activity of an enzyme (i.e. streptokinase) in an electrosprayed nanoparticles with minimum size and size distribution.

## Materials and Methods

### Materials

Purified recombinant streptokinase (without human albumin) was purchased from Pasteur Institute (Iran). PLGA (Mw ≈ 45 kDa) was purchased from Shenzhen EsunIndustrial Co. (China). The plasmin-specific substrate (S-2251) was purchased from Chromogenix (USA) and all other chemicals were obtained from Merck chemicals (Germany).

### Preparation of nanoparticles

PLGA was first dissolved in dichloromethane. Then, streptokinase was added to the solution at concentration of 0.05% w/v and stirred for 15 minutes at 4 °C. The concentration of enzyme was fixed for all the samples. Aqueous to organic ratio was considered 1:10 to minimize the effect of dichloromethane on structure and activity of SK. For ANNs Study, different values for applied voltage value (8-13 kV), nozzle internal diameter (needle ID, 0.18-0.34 mm) and ratio of polymer to enzyme (5-17) were investigated. The collecting distance (i.e. distance between nozzle and collector) and flow rate were fixed at 10 cm and 0.1 ml/h, respectively^11,21^ An emulsion containing SK-PLGA was pumped using syringe pump. Positive electrode was connected to needle and collector was connected to earth. By applying certain voltage, electrical force overcomes the surface tension of droplets that are produced at tip of the needle. So, a cone jet appears, followed by evaporation of the solvent during the jet process and formation of nanoparticles on the collector.^22^

### Artificial neural networks studies

In this research, ANNs software (INForm V4.02, Intelligensys, UK) was used in modeling the relations between inputs and outputs. Results from the generated model were illustrated as 3D graphs (i.e. response surfaces). Three factors were considered as input variables, including voltage (kV), polymer to enzyme ratio and diameter of the needle (mm). Average and standard deviation of size were considered as indicators of size and size distribution, respectively. 28 samples were prepared having random values for the above mentioned inputs.

From the different samples, 20 samples were taken as training set to train the network of relationships between the input and the output parameters. 2 samples were selected as test data to avoid overtraining of network and 8 samples were used as unseen data to validate the model. The training parameters used during modeling are shown in Table 1. Unseen data are shown in Table 2.

To validate the generated model, coefficient of determination (R^2^) of the unseen data was computed based on equation 1.^23^ A model with R^2^ value closer to unity indicates a better predictability.

(Eq. 1) R2=1−∑i = 0n(yi−y‵)2∑i = 0n(yi−y¯)2

Where n is the number of unseen data, y is mean of the dependent variable and ỳ is predicted value by the model.

### Particles size, morphology and zeta potential

To study the physicochemical properties of SK-PLGA NPs, a sample with PLGA and SK concentration (g/ml) of 0.5 and 0.05, collecting distance (cm) and needle ID (mm) of 10 and 0.18, flow rate (ml/h), applied voltage (kV) and polymer/enzyme and water/organic phase ratios of 0.1, 11, 10:1 and 1:10 was prepared. Morphology and manually calculated mean (standard deviation) particle size were investigated using scanning electron microscope (SEM, Hitachi, Japan) and zeta potential was studied with Zetasizer (Nano-ZS , Malvern Instruments Ltd. UK).

### Determination of yield, protein loading capacity and encapsulation efficiency

Yield was determined as the ratio between mass of dried streptokinase-loaded PLGA nanoparticles (SK-PLGA NPs) and total mass of used PLGA and SK.^24^

(Eq. 2) $$Yield\;\left(\text{\%}\right)=\frac{\text{mass of obtained SK}-\text{PLGA NP}}{\text{mass of used PLGA }+\text{ streptokinase}}\;\times 100$$

Encapsulation efficiency (EE) was taken as amount of protein that was encapsulated with respect to total amount of the protein, used for preparation of NPs. Determination of EE and loading capacity (LC) was evaluated by “non-entrapped” method proposed by Xu and Hanna:^24^

Particles were centrifuged at 12,000 g for 10 min. Supernatant containing free streptokinase was then assayed with Bradford assay.^25^ LC and EE were calculated according to Eqs. (3) and (4):

(Eq. 3) $$LC\;=\frac{A-B}{C}\times 100$$

(Eq. 4) $$EE\;=\frac{A-B}{A}\times 100$$

Where A is total mass of SK, B is mass of free SK and C is the total mass of particles.^24-26^

**Table 1:** The training parameters used with INForm v4.02.

**Output Parameters**	**Size**	**Size distribution**
Network structure		
No. of hidden layers	1	1
No. of nodes in hidden layer	3	3
Back propagation type	Incremental	Incremental
Back propagation parameters		
Momentum factor	0.8	0.7
Learning rate	0.8	0.7
Targets		
Maximum iteration	1000	1000
MS error	0.0001	0.0001
Random seed	1000	10000
Smart stop		
Minimum iterations	20	20
Test error weighting	0.1	0.1
Iteration overshoot	200	200
Auto weight	1	1
Smart stop	Enabled	Enabled
Transfer function		
Output	Tanh	Asymmetric sigmoid
Hidden layer	Asymmetric sigmoid	Tanh

**Table 2:** Unseen data that were used for validation of the model.

**Needle diameter (mm)**	**voltage (kV)**	**polymer/enzyme** **concentration (w/w)**	**Obtained size (nm)**	**Predicted size (nm)**	**Standard deviation (nm)**	**Predicted standard deviation (nm)**
0.34	10	15	72.5	68.1	18.1	17.6
0.18	8	10	43	41.1	12.1	11.1
0.34	8	10	55.7	56.9	17.5	16.7
0.26	12	5	32.0	31.7	8.2	11.8
0.18	11	10	37.0	36.0	12.1	15.5
0.26	13	7	39.5	32.4	9.6	11.8
0.26	9	12	48.0	44.9	16.9	16.1
0.34	9	17	75.0	68.1	18.5	17.7

### In vitro release

To study release profile, 2mg of NPs was dissolved in 1 mL PBS, and incubated at 37°C by stirring at 125 rpm. At certain intervals (i.e. 0.5, 1, 2, 4, 8, 12, 24, 48, 72 hours), 200 µL of the solution was removed and centrifuged (12000 g) for 10 min, then, the concentration of SK was assessed by Bradford method.^25^ The cumulative release (%) at each time point was determined using equation 5:

(Eq. 5) $$Cumulative\;release\left(\%\right)=\frac{cumulative\;release\;amount}{loading\;amount}\times 100$$

### Determination of SK activity

A colorimetric assay was used to evaluate the activity of SK. 1 mg NPs was added to 50 µL of fresh human plasma in 96 well microplates and incubated for 10 min at 37°C. 50 µL of 0.16% free SK was added to human plasma as control.^27^ Afterwards, 50 µL of 0.75 mM of S-2251 (D-Valyl-L-leucyl-L-lysine 4-nitroanilide dihydrochloride) was added to each well and incubated for 20 min at 37°C. 25 µL acid acetic 25% v/v was then added to stop the reaction. Absorbance of samples was read by microplate reader (biotek, USA) immediately at 405 nm against blank^28^ against PLGA NPs as blank. All experiments were done in triplicate.

## Results

### ANNs modeling

**Figure 1:**
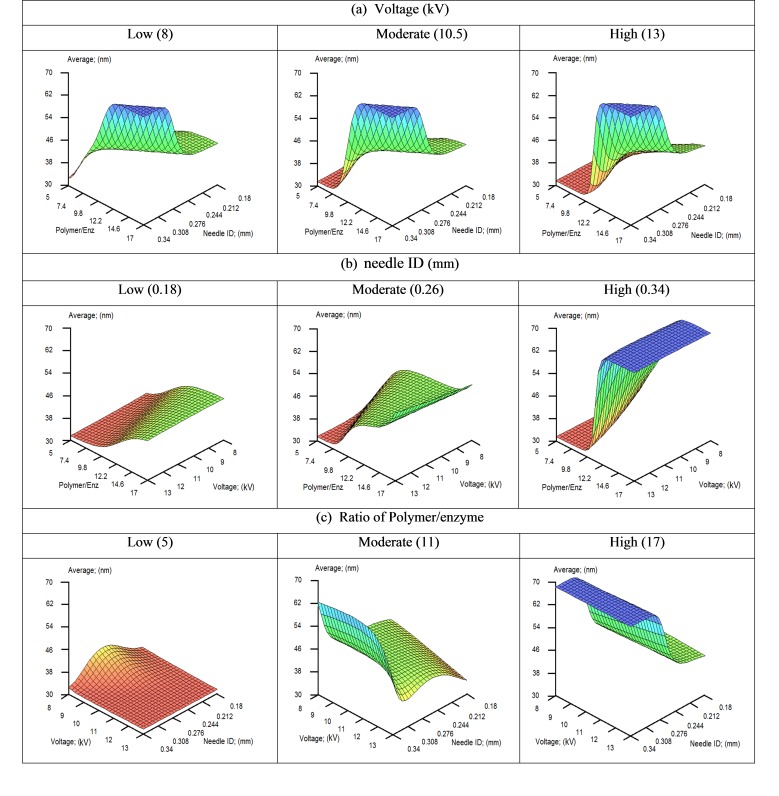
3D Plots of average size of nanoparticles predicted by the ANNs model when voltage (a), needle ID (b) or polymer/enzyme ratio (c) is fixed at low, medium and high levels.

**Figure 2:**
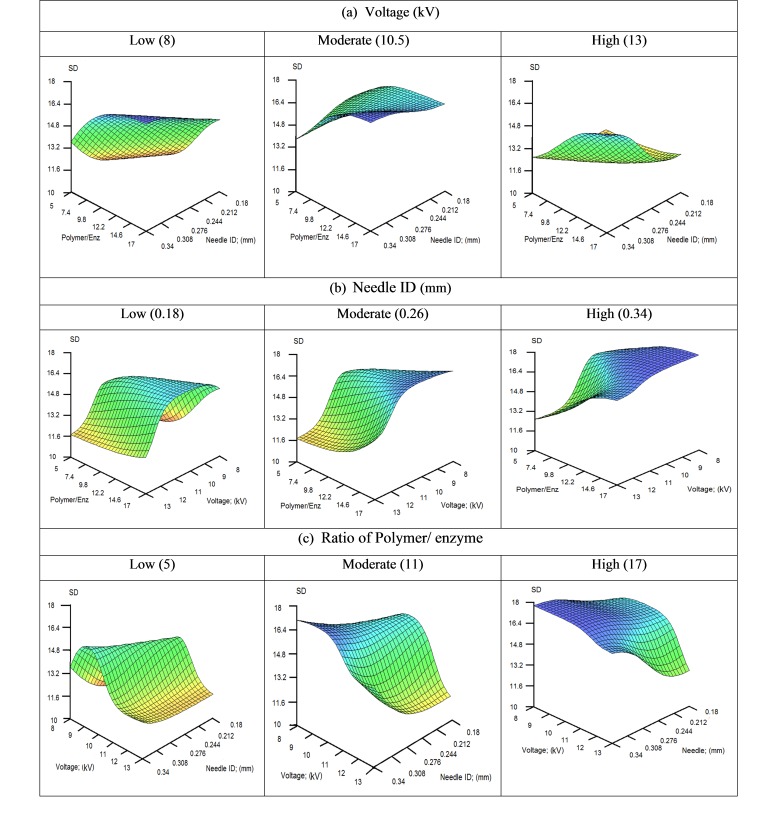
3D Plots of SD predicted by the ANNs model when voltage (a), needle ID (b) or polymer/enzyme ratio (c) is fixed at low, medium and high level.

The obtained models showed R^2^ values of 0.93 and 0.72 for the unseen data of size and size distribution (SD), respectively. The obtained values indicate satisfactory predictability for the models. Influence of three variables on the output through 3D graphs was investigated when a variable was fixed at low, moderate or high values. The influences of needle ID, ratio of polymer/enzyme and voltage on size and size distribution have been illustrated in Figures 1-2.

In Figure 1a, the effect of needle ID and ratio of polymer/enzyme is shown when voltage is fixed at high, moderate and low values. From the details, increasing either of needle ID and ratio of polymer/enzyme make increase the average size of nanoparticles.

In Figure 1b, needle ID is fixed and effect of ratio of polymer/enzyme and voltage on the size has been illustrated. Voltage does not appear to substantially influence the size but by increasing ratio of polymer/enzyme, size of nanoparticles is increased, especially at high values of needle ID.

As detailed in Figure 1c, which illustrates the effect of needle ID and voltage on the size, at low polymer/enzyme ratio, voltage and needle ID are not showing an important effect. While when concentration ratio is medium or high, by increasing needle ID, size of nanoparticles increases. Furthermore, in general, increasing voltage makes a slight reduction in size.

In Figure 2, an input parameter has been fixed at a low, medium or high value to visualize the influence of other two parameters on the output. The Figure 2a, shows the effect of polymer/enzyme ratio and needle ID on the SD, when voltage is fixed. From the details, a small increase in SD may be observed by increasing either of ratio of polymer/enzyme or needle ID.

In Figure 2b, the influence of voltage and polymer/enzyme ratio on size distribution is shown when needle ID is fixed. The details show that in general, SD is increased by decreasing of voltage. Exceptionally, when needle ID and polymer/enzyme ratio are both low, very low values of voltage make the SD smaller.

Figure 2c shows changes in size distribution as a function of needle ID and voltage when polymer/enzyme ratio is fixed. As mentioned above, the main observation is increasing of SD when voltage decreases with an exception in low values of all the three parameters. As this particular point of the graphs is not following the other rules, it appeared to us that a local defect in model training was the reason for this observation. We therefore excluded this particular point from our findings.

### Release studies

**Figure 3:**
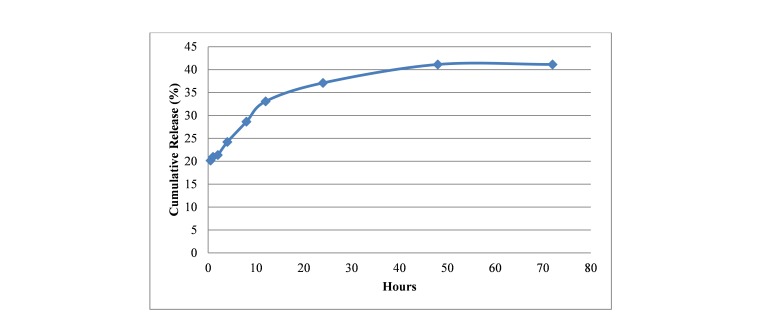
Release profile of SK from PLGA-SK NPs at different hours.

In the Figure 3, profile of drug release at specified intervals (i.e. 0.5, 1, 2, 4, 8, 12, 24, 48, 72h) is illustrated. A burst effect may be observed from the figure, followed by a steady increase for the first ~40 hours.

### Size, morphology and zeta potential of nanoparticles

**Figure 4:**
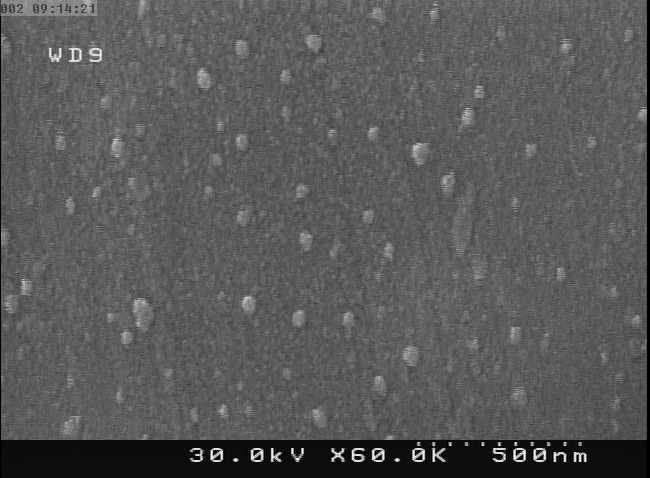
SEM image of Streptokinase -loaded PLGA nanoparticles

Size, size distribution were investigated by SEM (Figure 4), giving mean (standard deviation) of 37(12) nm. Also, particles showed a zeta potential of -43.6 (mV).

### Activity of SK

**Figure 5:**
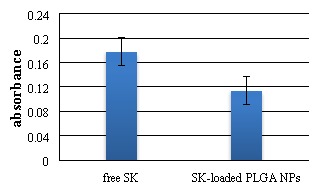
Absorbance of S-2251 in SK-loaded PLGA NPs and free SK after 30 minutes.

According to SK activity assay, absorbance of S-2251 in SK-loaded PLGA and free SK was 0.114 and 0.178 (see Figure 5), respectively. This shows that only 19.2% of the SK activity has been preserved during the electrospray process.

### Encapsulation efficiency and loading capacity

The yield, EE and LC of optimum sample was determined as 86%, 90% and 8.2%, respectively.

## Discussion

The process of electrospray is classified into two principle categories: dripping and jet modes. Dripping mode is consisted of fragments of solution that are ejected directly from the needle with shape of regular large drops. Dripping mode may be divided into microdripping (fine drops) and spindle (elongated drops) modes. Irregular fragments of solution may also be generated. At dripping mode, when applied voltage to the nozzle increases slowly, droplets become smaller and number of generated droplets increases which is the start of microdripping mode.^29^ In microdripping mode, particles are usually smaller with regular shape.^30^ In jet mode, solution is stretched along the capillary axis to form a regular and thin jet that is named cone-jet or Taylor cone.^29^ The cone-jet mode is a stable mode which generates smaller and uniform droplets.^30^ Shifting the jet mode to the dripping mode has been reported by decreasing viscosity, applied voltage, collecting distance and surface tension as well as increasing needle ID.^31,32^ Although, in principle it is expected that changes in jet mode leads to changes in size distribution, works which have experimentally examined that are very limited as described above.

Equation 6 provides the effect of electrospray factors on size of produced nanoparticles:

(Eq. 6) $$r=\sqrt[{3}]{\frac{3}{2\rho g}\left[r_{0}\gamma -2\;\varepsilon_{0}{\left(\frac{V^{2}}{ln\left(\frac{4H}{r_{0}}\right)}\right)}^{2}\right]}$$

Where r is radius of droplets that are formed during electrospray, ρ is density of solution; g is acceleration due to gravity, r0 is theradius of needle, γ is surface tension, ε0is the permittivity of air, V is applied voltage and H is the collecting distance.^31^

From our findings, to obtain the smallest size, ratio of polymer/enzyme should be low. Since the enzyme concentration was fixed during our work, change in polymer/enzyme ratio was a function of polymer concentration. Increase in concentration of polymer solution leads to increase in surface tension and viscosity and decrease in electrical conductivity which generate bigger particles.^33,34^ The results agree with pervious study that used PLA-BSA emulsion in electrospray. The report shows that increasing the concentration makes the size larger.^34^ Also, decreasing the needle ID caused a decrease in size of generated nanoparticles, probably due to generation of smaller sprayed droplets which upon drying produce smaller particles. However, this finding appears not to be in agreement with some other works. For instance, in preparation of chitosan nanoparticles by electrospray, needle ID showed no important effect on size of particles.^17^ Also, in electrospray of polycaprolactone, particle size when comparing needle gauge (G) of 21 with 26, average size was not importantly different.^12^ We also found that voltage is only slightly affecting the size. At first it appears that this finding does not agree with the well-known fact that voltage is a key factor in determining the size of nanoparticles in electrospray.^35^ However, it is already reported that by decreasing the flow rate from 3 ml/h to 0.12 ml/h, the effect of voltage becomes considerably,^35^ a fact which could explain our finding.

The findings also showed that to obtain smallest SD, voltage should be high. Increasing voltage could overcome surface tension produce a stable jet mode. So, SD decreases. Previous report showed that increasing the voltage lead to decreasing the SD.^30^ Higher voltage probably leads to generation of jet mode in electrospray which makes size distribution lower compared with the dripping mode.^36,37^

Our results showed that increase in needle ID or polymer/enzyme ratio made a small increase in SD. Previous studies show that flow rate could directly affect SD of electrosprayed NPs,^17,38^ which is in turn directly affected by needle ID. A previous report on a limited number of data has reported that when polycaprolactone was electrosprayed with needle gauges 21and 26, size distribution was a little broader for 21.^12^ The effect of polymer/enzyme ratio in our work could be explained by the fact that the enzyme was fixed in all our experiments. Thus, increasing the ratio was due to increase in polymer concentration. In electrospray, electrical force is applied to overcome the surface tension and break the droplets to form smaller particles at the tip of needle.^34^ Increasing the concentration leads to increase of viscosity which is working against the effect of voltage in reducing SD.^39^

In this study, results of SEM showed nearly spherical particles with average size of 37±12nm. While other studies that used electrospray to encapsulate a protein in a polymeric particle, size was 22 micrometer,^21^ 20 micrometer,^20^ 1-4 micrometer^24^ and 4-5 micrometer.^34^ Considerably smaller sizes in our work were most probably because of diluted solutions that we used. In our study concentration of PLGA was 0.5% and ratio of PLGA/SK was 10:1. Whereas, concentration of polymer and ratio of polymer/protein were 6% PLGA and 10:1,^21^ 10% PLGA and 1:5,^20^ 3%PLA and 2-6:1^24^ as well as 3% PLA, 5:1,^34^ respectively. Another report, electrospraying PLGA and N-Acetylcysteine (not protein), size of particles was 122 nm with 0.5% of PLGA,^19^ similar to ours.

High encapsulation efficiency in electrospray is the main advantage of this method which has made it attractive for many researchers.^12^ EE in this work was 90%, comparable with other electrospray works (e.g. 76%,^20^ 80%^24^ and 82%^21^). This value is essentially higher than other methods to encapsulate proteins in a polymer. For instance, Modarresi *et al*.^40^ added SK solution to chitosan under stirring and obtained encapsulation efficiency of 64%.

During the electrospray of the dispersion containing SK and PLGA, SK was encapsulated in PLGA nanoparticles. When SK-PLGA NPs are in contact with PBS, diffusion of water in surface of particles causes release of SK in medium. The results show that almost 20 percent of the drug was released during the first 30 minutes and the release study continued for about 72 hours. During this period, approximately 41% of the initial concentration of the enzyme was released. In another study, BSA was encapsulated in microparticles of PLGA by electrospray method. Release of BSA in the first 24 hours was almost 30% and after 6 weeks, release was 50-60%, diffusion of water in particles and release of BSA at surface of particles caused burst effect.^21^ Our results indicated that the electrosprayed nanoparticles of SK have lost its activity considerably, contrary to what we expected. Instability of protein during production process or in contact with organic solvents is an important problem for using them in industry.^41^ In an electrospray process, there are parameters which potentially damage a protein’s activity: aggregation of protein as well as unfolding and degradation of protein in organic/aqueous interface.^42^ These two phenomena are possibly the most important reasons for the decrease in activity. It should also be noted that 80% of the enzyme does not release during the first 30 minutes. Thus, most of the enzyme molecules are out of reach of the substrate (except those who have been released or are on the surface of the nanoparticles), another possible mechanism which reduces activity of the enzyme.

## Conclusion

The main goal of this study was to produce monodispersed nanoparticles of PLGA-SK by electrospray and evaluate their activity. Polymer/enzyme ratio olymer/enzyme ratio and voltage were found to be dominant parameters in determining size and size distribution of the nanoparticles, respectively. The optimum preparation showed mean (standard deviation) size, yield, encapsulation efficiency, loading capacity of 37 (12) nm, 86%, 90% and 8.2%, respectively. The enzyme encapsulated in the nanoparticles however showed sharp decrease in its activity.

## Acknowledgments

This research has been supported by Tehran University of Medical Sciences‏ & health Services grant No 93-04-87-27550

## Ethical Issues

Not applicable.

## Conflict of Interest

The authors declare no conflict of interests.
